# Progress of Veterinary Science

**Published:** 1881-07

**Authors:** 


### PROGRESS OF VETERINARY SCIENCE.

---

Vegetable Charcoal is highly recommended by English army veterinarians as a cheap and efficient dressing for wounds.

Gout in Parrots has been observed by M. Meguin, even when the birds were kept upon a vegetarian diet. This is a fact which is rather hard to explain upon the accepted theories of the pathology of gout.

A Case of Splenic Leucocythemia in a pig is reported by M. Henley, M. R. C. V. S., Veterinary Department, Ireland. The case was not diagnosed before death, and it is imperfectly reported in the *Veterinary Journal.*

Cases of Fibrinous Endocarditis in a Boar, and in a Cow, with resulting mitral obstruction, are reported by R. Roberts, M. R. C. V. S. The animals showed signs of distension of the venous system, with dyspnoea and weakness.

A Case of Rabies in the Horse was recently reported by J. C. Berne, of the Irish Veterinary Department, in the *Veterinary Journal.* The animal had been bitten in the lip by a rabid dog, two months before. The horse showed signs of the greatest nervousness and prostration, but there were few convulsions, and there was no aversion to water or inability to drink it.

Iodoform in Ozœna, Nasal Gleet, and Skin Diseases, is highly recommended by some English veterinarians. Mixed with two parts lycopodium, one of tannic acid, and blown upon the nasal mucous membrane, it acts very quickly and successfully. In painful ulcers it has an anodyne and antiseptic effect. In some of the skin diseases of dogs, given in doses of three and five grains, it is very useful.

The Red Blood Globules in the dog and horse and domestic animals generally, number 7,500,000 per cubic centimeter; those of man 5,000,000. In the domestic animals the proportion of white to red varies from 1 to 800, 900, 1,000 or 1,200. In the case of leucocythemia it is diminished to 1 to 85, 1 to 50, 1 to 15 or even 1 to 12; but it does not get to the proportions found in human leucocythemia, *i. e.*, 1 to 2, 2 to 3.

The Preservation of Anthrax Poison in Soils.—In connection with the note on animal poison on next page, it will be remembered that Pasteur advanced the theory that anthrax germs were always preserved in the soil for a long time. They might be brought to the surface earth by the earthworms, and thus made to affect animals grazing over the place. This assersion of Pasteur was denied by Mr. Colin. A Commission appointed by the French Academy of Medicine to investigate and decide the question, recently made a report in favor of Pasteur's theory.

A New Cestoid Parasite.—At a late meeting of the Paris Biological Society, Dr. Megnin exhibited a specimen of a new parasite, Triscuspidaria nodulosa, which presents many marked differences from the ordinary tænia, having claws in place of suckers on the abdomen, and pursuing all its generations in the same animal. The parasite is found in the perch, trout, sunfish and some other fish, and it is not impossible it may be at times found in man.—*Chicago Medical Review.*

Trichinæ in Chickens.—Cobbold and others have fed trichinous flesh to poultry, and afterwards examined the flesh for trichinæ, but in no case were any found. This is a singular circumstance, for which we believe no satisfactory reason has thus far been adduced. At first sight, one might suppose that the peculiar mode of digestion which obtains in the case of birds might be the cause, but this is evidently not the true explanation, since living trichinæ are found abundantly in the lower part of the digestive tract after the fowl has been fed with trichinous flesh. Thus far, however, we believe that no trichinæ have ever been found encysted in the flesh of the ordinary breeds of poultry.—*American Journal Micros.*

A Canine Epileptic Affection Caused by Acari.—Meguin recently communicated to the Societé de Biologie, the existence of an epileptiform affection, apparently contagious, which decimated for several years the kennel of a rich dog fancier near Havre. On careful examination, it was found that the disease was due to the presence of an acarus in the auditory canal of the dog. This acarus, already known under the name of *Chonoptes Ecaudatus*, has been found in the dog, cat, and ferret, but only in the cat has it been recognized as the origin of a vertiginous affection. Injection of an aqueous, one in five, solution of sulphate of potash cured the disease. —*Chicago Medical Review.*

The Persistent Effect of Animal Poisons.—That germs of disease may live long, seems to be proved by recent experiments of a French Committee directed by M. Pasteur. Seven sheep were led daily, for a few hours, to a piece of ground where some animals that died of anthracoid disease, or *charbon*, had been buried *twelve years previously.* Two of them caught the disease and died. There was no grass for the sheep to eat, and it is thought those two must have received the germs in the course of smelling about the ground, as sheep generally do. It may be added that on some ground of that farm, where diseased animals had been buried, vegetables are now grown; and M. Pasteur asked the farmer if any employes, etc., about the farm had been affected. He knew of none, but showed a healed sore of malignant pustule (the same kind of disease) on his own face. M. Pasteur supposed that if the vegetables eaten had not been cooked, there might have been a different tale. The disease, too, might be communicated by the agency of insects, etc.

The Effect of Lightning upon Animals.—Lightning, according to Mr. G. A. Banham (*Veterinary Journal*), strikes the lower animals more powerfully and oftener than it does man. Some examples of this are given. During a thunder-storm in France, 78 sheep and 2 dogs, watching them, were killed at one flash, the woman in charge being only slightly affected.

A man was stunned by lightning which killed a cat beside him.

Many instances are given in which horses, mules or oxen have been killed, while the driver was but little injured.

Another fact is, that when animals are herded together, or are standing in a line, those at the outside are injured most.

White-haired animals seem to be oftener struck.

As to the lesions of a lightning stroke, it is calculated that only in about one-sixth of the cases are any anatomical changes to be found. These changes are generally in the nature of burns, rupture of muscles, or external marks of some kind, perforations, fractures, etc. Rigor mortis is usually well marked.

The metal shoes of the animals may be magnetized.

Death is produced usually by shock. If death does not occur at once, the animal usually recovers rapidly.

It is an important fact, from a medico-legal point, to remember that an animal may be killed by lightning and show no trace of the cause of death.

THE RATION OF ENSILAGE FOR A COW.—Some experiments made at the New Jersey Agricultural Experiment Station have thrown much light upon the value of ensilage. Concerning the ration for a cow of 1400 lbs. weight the following is stated: If she is a good milker and the ensilage is green corn, she will require 90 lbs. daily of corn ensilage, or 16,380 lbs. in six months; and besides this, say 8½ lbs. of corn meal and 8 pounds of bran mixed per day. Let us put this in the formula of a ration:

RATION FOR A 1400 LB. COW IN MILK.

| | *Dry substance, lbs.* | *Albuminoids, lbs.* | *Carbo-hydrates, lbs.* | *Fat, lbs.* |
|---|---|---|---|---|
| 90 lbs. of ensilage | 17.37 | 1.08 | 9.00 | 0.41 |
| 8½ lbs. of corn meal | 7.40 | 0.64 | 5.72 | 0.26 |
| 8 lbs. of bran | 7.09 | 0.80 | 3.88 | 0.25 |
| | 31.86 | 2.52 | 18.60 | 0.92 |

Nutritive ratio 1:8.3.

This gives food enough, but the proportion of albuminoids is too small. If we substitute 2½ lbs. of linseed meal for that amount of corn meal, the amount of albuminoids will be over 3 lbs. per day, and will do very well.

TRANCE IN THE LOWER ANIMALS.—In connection with Dr. Beard's notable article on this subject in our last issue, we publish the following account by Mr. Carl Orchsemius in *Nature:*

He says: In the interior of the province of Valdivia, South Chili, a species of wood snipe is often caught by the natives in the following manner: When the bird flies into one of the low bushes, two men on horseback go around it in the same direction, swinging meanwhile their lazos over the bush. After ten or more rounds one man slips down from his horse, while the other continues to ride round the undergrowth leading his companion's horse. The dismounted rider carefully creeps on to the place where the snipe sits in a state of stupor and nearly motionless from the effect of the rider's circular movements, and kills it with a quick blow of a stick. When the writer was first told of this mode of capturing a bird, he would not believe it, but he himself, in 1853 or 1854, took part in this very sort of method of taking the bird in the hacienda San Juan, in Valdivia. He left the house without a gun. He saw a snipe fall into a dense bush, and expressed his regret to a servant that he had not a gun, so that he might secure a good specimen of a not very common species for the natural history collection. "Never mind," said the servant, "if you wish, we will get the bird;" and the bird was caught, without injuring it, substantially in the way above described.

Diseases of Animals in Captivity.—The following interesting list of the causes of death of animals in the Phialdelphia Zoological Gardens is sent us by Mr. Arthur Erwin Browne, the Superintendent:

| | Name of Animal. | Cause of Death. |
|---|---|---|
| 1. | *Siren lacertina* . . . . . | No organic disease. |
| 2. | Moor Monkey (*Mac. maur.*) . . . | Phthisis. |
| 3. | Howling Monkey (*Mycetes pall.*) . . | Heart disease. |
| 4. | " " " " . . | Inflammation of intestines. |
| 5. | Ant Eater (*Myrmecop. jub.*) . . . | No organic disease. |
| 6. | Howling Monkey (*Myc. pall.*) . . | Phthisis. |
| 7. | Cassowary (*Casuar. gal.*) . . . | Dysentery. |
| 8. | Porcupine (*Hystria crist.*) . . . | No organic disease. |
| 9. | Phalanger (*Phalan. mep.*) . . . | Peritonitis. |
| 10. | Porcupine (*Hystria crist.*) . . . | Heart disease. |
| 11. | Sea Lion (*Zal. gillesp.*) . . . | Tuberculosis. |
| 12. | Phalanger (*Phal. mep.*) . . . . | Paralysis. |
| 13. | Kangaroo (*Petrogale pencil.*) . . . | Hydatids of liver. |
| 14. | " " " . . . | No organic disease. |
| 15. | *Menobranchus lat.* " . . . | Taenia and Peritonitis. |

Farcy.—Farcy is a strictly contagious disease, but it is not so easily communicated from the diseased to the healthy animal as glanders is. Among the means by which farcy is most readily communicated may be mentioned the use of the same harness, blanket, brush and curry-comb, on other horses.

It may develop spontaneously; that is, as a result of faulty ventilation, excessive labor, together with insufficient and unsound food; in fact, from any cause that tends to reduce the vitality of the system. It is frequently a sequel to chronic diseases of various kinds, both internal and external.

In appearance it bears no resemblance to glanders, though it bears a certain relation to it; in fact, farcy and glanders may be regarded as twin diseases.

Either of the two diseases generally terminates both forms fully developed.

The treatment of farcy is not often successful, and requires considerable patience and judgment, which the amateur cannot be expected to possess. We have always hesitated in recommending any treatment of farcy, because the remedies from which the greatest measure of success is likely to be obtained, such as a combination of strychnine with arsenic, phosphorus, etc., are too heroic in their effect, and too dangerous to be trusted in the hands of novices. The physiological effects of such remedies must be closely watched to prevent fatal accidents. The necessity of gradually increasing the doses, and of diminishing or entirely withholding them, is another consideration. Again, the long continued use of any remedy has a tendency to lessen its effect, from the system becoming, as it were, habituated to them. This renders the alternate use of such remedies a necessity. Treatment of farcy can only be recommended in the beginning of the disease, and, with that understanding, the following remedies may be used: To the farcy ulcers apply either a strong solution of chloride of lime, carbolic acid, or lunar caustic; or touch with hot pointed iron. Internally give, twice daily, half a drachm of iodide of copper, or iodide of iron, with half an ounce of gentian root. An ounce of aloes, with half an ounce of saltpetre, may be given every fortnight. Keep salt constantly placed within the reach of the horse. Give good nourishing food, in liberal quantity, daily exercise, or liberty on pasture, and attend to general cleanliness. Thorough disinfection of premises and utensils is necessary.—*Dr. Paaren, N. L. A. Jour.*

Dropsy in Horses.—From the *National Live Stock Journal* we learn that dropsy in horses is unusually prevalent the past season, excited by the sudden and extreme changes in the temperature.

Plethoric animals, and those in an anæmic condition, seem to be the chief sufferers. For this diseased condition preventive measures are recommended. In the stable, attention to cleanliness, dryness, good ventilation and sunshine—to all those things which tend to maintain sound and vigorous health. Exposure to severe storms or sudden changes in temperature are to be avoided, as far as possible, and mitigated by suitable clothing; especially are they to be avoided during the spring and autumn, when changes in the coat are occurring. "All disorders of the air-passages calculated to impair the condition of the blood, and all debilitating diseases, should be attended to at the outset, especially in damp, cold, and exposed localities, where the tendency to these dropsies is the most marked." "For the treatment of those diseases which affect the air-passages all depleting measures are to be avoided." "The patient to have warm, dry, comfortable buildings, with the purest air, sunshine, and soft, nourishing, and easily digested food." "If there is any tendency to coldness of the surface and limbs, the body should be warmly clothed, and the limbs loosely bandaged, to avoid those chills and nervous derangements which are so likely to cause dropsical complications." For the treatment of the dropsy the following is recommended: First, an aloetic purge, followed by a daily dose of nitrate potassa, half ounce; iodide potassa, one drachm; chlorate potassa, half ounce; nux vomica, ten grains; and gentian, two drachms.

The swellings to be rubbed with a liniment composed of turpentine, one ounce; sulphate zinc, half ounce; olive oil, one pint; and if upon the belly or legs, scarifying is admissible, but to be avoided if upon the nose, where sloughing is likely to occur. If the patient is anæmic, the purge must be dispensed with, and stimulants and tonics administered, with good nursing.

Rupture of the Horse's Stomach.—Rupture of the stomach is more common in the horse and ox than in all other animals. It is produced in various ways. Cattle have a certain carelessness regarding the ingredients of their diet, and frequently swallow with food all sorts of foreign bodies, such as nails, forks, knives, pieces of iron and bone, &c. These occasionally lacerate the stomach. Cattle and horses turned into new clover, or luxuriant pastures—after having been kept on dry food—very frequently die from rupture of the stomach as a consequence. They eat ravenously until their stomachs are over-distended; the material is rich and succulent, and not properly masticated, and ere the gastric fluids can act upon the crude mass and bring about digestion, fermentation takes place; carbonic acid gas is largely eliminated, which inflates the stomach, and if produced in sufficient quantities, the stomach is literally blown to pieces. Horses fed on Indian corn, cut feed, or after a change of diet, are especially liable to indigestion, fermentation and rupture of the stomach. Horses with tympanitis (bloat colic) often rupture themselves in falling during the paroxysms of pain. I have frequently known horses to be well at noon after the morning's work, eat their dinner, and tympanitic colic kill them in three hours, either by mechanical pressure or rupture. Another cause in the horse is one which would not be so apt to arise in other animals—the horse's stomach is small in proportion to his size. Now, if he is over-fed or gets loose and gorges, and then gets to water, or is allowed to drink, the swelling of the food may produce rupture. Any slip or exertion has a like result.

The symptoms are uneasiness, pain, stretching out the forelegs, arching the back, walking round and round, endeavoring to get the fore-legs higher than the hind-legs (as to stand-point), and sitting up on the haunches like a dog; agony is written on the anxious countenance; cold sweats and tremors of the limbs and muscles are perceptible. Vomiting is a symptom of rupture in the horse, and dark fluids issue from the nostrils on some occasions. The pulse is quick, weak, and soon becomes imperceptible.—Dr. E. MOORE in *Country Gentleman.*

LAWS RELATING TO CONTAGIOUS DISEASES OF ANIMALS IN GERMANY.—An elaborate series of provisions has recently been made in Germany for preventing the spread or importation of contagious diseases among animals. The part relating to the suppression of the disease is as follows:—

The owner of domestic animals is obliged to make immediately to the police authority the declaration of the appearance amongst his stock, of one of the epizootic diseases, as well as of the suspicious signs making him fear the existence of such disease, and to keep the animal away from the places where there is danger of other animals being contaminated.

These same obligations exist for the agent of said owner, and also in relation to the animals in transportation to those who have them in charge, and in relation to animals given in keep to other parties, to the owner of respective farms, stables, pastures, &c.

Are also obliged to make the same declaration the veterinary surgeons and all persons which professionally exercise veterinary medicine; as well as the inspectors of slaughter houses, as well as those whose profession is to remove, sell or work on animal cadavers or substances when, before the police have taken measures, they have knowledge of the eruption amongst animals of one of the following epizootic diseases or the apparition of suspicious signs.

The epizootic diseases to which the obligatory declaration is applied are:

1st. Anthrax.

2d. Hydrophobia.

3d. Glanders and farcy of horses, donkeys, mules.

4th. Foot-and-mouth dieases of bovine, sheep, goats and swine.

5th. Pleuro-pneumonia of bovine.

6th. Small-pox in sheep.

7th. Maladie of coit (dourine) of horses and the coital exanthems of horses and bovines.

8th Scabies of horses, donkeys, mules and sheep.

The Chancellor of the Empire is authorized to make also for other epizootic diseases the declarations transitorily obligatory.

For the districts where anthrax exists permanently, the governments of the different States of the Confederation are authorized to take it off the obligatory declaration as long as the disease appears only on isolated cases.

ROUND WORMS IN THE ALIMENTARY TRACT OF SHEEP.—Three varieties are mentioned, and declared equally as dangerous as the tape-worm.

First, the *Strongylus contortus*, a white cylindrical worm, from one to two inches long, tapering equally at both ends, which inhabits principally the fourth stomach.

Second, the *Strongylus filicollis*, from one to two inches long, but furnished with a long tapering neck and round mouth, which inhabits the small intestines.

Third, the *Trichocephalus affinis*, or *whip-worm*, a round worm, but smaller than the two preceding. This variety is only six or eight lines long, the anterior part of the body attenuated to a fine hair, and inhabits the blind gut and colon. The three varieties are found clinging to the mucous membrane in great numbers if the animals are opened soon after death. Later they become detached, and are often overlooked. They produce death both by sucking the blood from the animal, and keeping up a constant irritation. The *Strongylus filicollis* is said to be the most destructive of the three, for two reasons: First, the location, and, second, the formation of the mouth, enabling them to produce more irritation. The *Trichocephalus affinis* is often found with one end deeply imbedded in the mucous membrane, but it is the least irritating, probably because the colon is the least sensitive part of the alimentary tract. In small numbers, all three varieties are harmless, but when present in large numbers they are very destructive, and especially to the young of a flock.

The same general symptoms are present in all the varieties—loss of condition, spirits and vigor, tenuity and lifelessness of the skin, pot-belly and razor back, irregular appetite and fœtid diarrhœa. The diarrhœa is the marked symptom, and often, by carefully examining the fæces, some of the worms can be found.

These parasites differ from the tape-worm in not passing through a series of changes in different animals, but develop upon the receipt of the egg into the alimentary tract of the sheep. The embryo worm is as tenacious of life as the Tænia, maintaining its vitality in water, moist earth, on green vegetables, but only rapidly developing when introduced into the sheep. One male and female are all that is needed to produce millions.

Owing to this great tenacity of life, land may become infested with the eggs, and successive flocks destroyed. Fodder cut from infected land can be fed with impunity to horses and hogs, but is dangerous to the sheep.

The succulent and juicy crops harbor the most prolific eggs. The eggs also appear most prolific when the dew is on the grass, and in the springtime, when the sheep tear up roots and all. If infected pastures must be used, dry days only should be chosen.

Surface drainage may be the cause of a well becoming impregnated, and in that way whole flocks be attacked. Streams running through infected pastures may be a cause of spreading the parasites.

A high-priced ram may come into a flock with a few in his system, but not enough to affect his condition; but by breeding and multiplying, they enter the weaker and more poorly fed, and young—constantly increasing in a geometrical ratio, until the hoped-for improvement of the flock by a new ram has resulted in the opposite—death of large numbers, with infected pastures.

At first the older sheep seem to suffer comparatively little, while the lambs melt away like snow, until a whole flock may be destroyed, and the lambs often become infected before the real danger is fully recognized and appreciated.

To rid the sheep of the parasite, ten grains of tartar emetic and ten grains of carbolic acid are recommended; also, oil of turpentine, one-half ounce; castor oil, four ounces, twice a week, salt to like, and bitter tonics.

Treatment should be conducted in yards which can be cultivated, and not used for pasturage until the disease has been arrested and completely exterminated. When the malady is arrested the animals should be turned into non-infected pastures. Infected land should always be cultivated for a long term of years.—*National Live Stock Journal*, March, 1881.

